# Micelle Mediated Trace Level Sulfide Quantification through Cloud Point Extraction

**DOI:** 10.1100/2012/152016

**Published:** 2012-04-24

**Authors:** Samrat Devaramani, Pandurangappa Malingappa

**Affiliations:** Department of Studies in Chemistry, Central College Campus, Bangalore University, Dr. Ambedkar Veedhi, Bangalore 560 001, India

## Abstract

A simple cloud point extraction protocol has been proposed for the quantification of sulfide at trace level. The method is based on the reduction of iron (III) to iron (II) by the sulfide and the subsequent complexation of metal ion with nitroso-R salt in alkaline medium. The resulting green-colored complex was extracted through cloud point formation using cationic surfactant, that is, cetylpyridinium chloride, and the obtained surfactant phase was homogenized by ethanol before its absorbance measurement at 710 nm. The reaction variables like metal ion, ligand, surfactant concentration, and medium pH on the cloud point extraction of the metal-ligand complex have been optimized. The interference effect of the common anions and cations was studied. The proposed method has been successfully applied to quantify the trace level sulfide in the leachate samples of the landfill and water samples from bore wells and ponds. The validity of the proposed method has been studied by spiking the samples with known quantities of sulfide as well as comparing with the results obtained by the standard method.

## 1. Introduction

Sulfide is one of the most essential parameter in monitoring the water quality due to its high toxicity for aquatic life. Dissolved sulfide is toxic to some fresh water fish even at the concentration level less than 1 ppm [1]. The threshold limit value of sulfide in drinking water has been prescribed as 0.05–0.1 ppm by the world health organization (WHO) [2]. Sulfide anion is an important constituent of aqueous systems wherever microbial colonies flourish. Because of its toxicity and removal ability of dissolved oxygen, there are limits on the total level of sulfide permitted in waste discharges [3]. Hence, its concentration needs to be controlled especially in water and waste water discharges. Monitoring of aqueous sulfide is one of the important parameters in industrial effluents as the discharges containing sulfide cause the contamination. The treatment of such water generally involves aeration, chlorination or flocculation, and other processes [4]. The treated effluent must meet regulatory specifications before it can be discharged into environment. Thus, it is mandatory to determine sulfide levels during various stages of treatment [5] Although sulfide is currently regarded as a core parameter for assessing environmental quality of a given water system, the clean matrices such as mineral water, biogenic sulfide in natural waters require highly sensitive methods to quantify trace level sulfide with high precision and accuracy [1]. Determination of sulfide in the waste water or the first-stage effluent is a challenging problem due to the complex nature of the matrix. Both petroleum- and kraft-processes-based paper and pulp industries generate sufficient quantities of sulfide bearing waste water. Sodium sulfide is one of the most widely used reagents for the removal of hair from animal hides with processing liquors possessing sulfide ion concentration up to 2000 ppm [6]. Sulfide gets released into aquatic environments substantially through bacterial mobilization of sulfur containing minerals.

Several methods have been reported for the quantification of dissolved aqueous sulfide. These are mainly based on titrimetric, chromatographic, and spectroscopic techniques. Among these, spectroscopic-related methods have been proved to be important due to its simplicity, selectivity, and sensitivity. Methylene blue method is considered as a standard method for the quantification of sulfide due to its sensitivity and easy adoptability. But the method suffers from the interference of thiols, and acidity plays a critical role in the production of phenothiazinium dye.

Several methods have been reported by adopting the same reaction using different analytical techniques like flow injection analysis, solid-phase extraction, and solid-phase reflectometry [7, 8]. In addition to the spectrophotometry, various other techniques like atomic florescence spectroscopy (AFS) [9], atomic absorption spectroscopy (AAS) [10], flow injection analysis (FIA) [11, 12], and inductively coupled plasma atomic emission spectroscopy (ICPAES) [13] have been successfully utilized for the quantification of sulfide. These techniques require expensive instrumentation and skilled technical personnel to operate the systems. Hence, spectrophotometric methods find widespread use due to their simplicity and easy operation in the estimation of sulfide. Organic solvents have been extensively used for the extraction of the metal-ligand complexes in spectrophotometric methods to lower the detection limit and to improve the sensitivity. However, the use of organic solvents is not preferred in recent years due to green protocols as they are toxic in nature. Hence, surfactants have been used as alternative reagents to enhance the sensitivity of the reaction as well as analyte preconcentration in cloud-point-based extraction procedures.

The combination of pronounced hydrophobic and hydrophilic properties within a molecule gives surfactant molecules unique properties on dissolution in water [14]. Surfactants on dissolution in water form organized molecular assemblies called micelles if the critical micelle concentration (CMC) exceeded. Organized molecular assemblies have potential utility in chemical analysis because micelles enhance the solubility of organic compounds in water by providing local nonpolar environment [15]. Watanabe and coworkers have introduced cloud point extraction (CPE) as a promising new separation and extraction technique as an alternative in place of organic solvents [16]. CPE based on the clouding phenomenon of surfactants has become attractive because it offers many advantages over traditional liquid-liquid extraction methods. Recently, cloud point extraction of ethylene blue for the estimation of sulfide in water samples has been reported [17].

Neutral surfactants have been extensively used in sensitizing the reactions in various analytical applications. However, use of cationic surfactant as a sensitizing agent has not been explored fully in recent years [18–20]. Here, we are reporting the use of cationic surfactant, that is, cetylpyridinium chloride (CPC), as a sensitizing agent to quantify trace level sulfide by its reaction with ferric iron and its subsequent complexation of reduced ferrous iron with nitroso-R salt to form a green-colored complex. The complex has been quantitatively extracted into surfactant-rich phase in the presence of potassium iodide. The method has been applied for the quantification of dissolved sulfide in the leachate, bore well, and pond water samples, and the results obtained by the proposed method are in good agreement with the standard methylene blue method [21].

## 2. Experimental

### 2.1. Apparatus

Absorbance measurements were made using Shimadzu Scanning Spectrophotometer (model UV-3101PC) using 1 cm quartz cuvettes. All pH measurements were carried out using a control dynamics digital pH meter (Model APX 175). All reagents were analytical grade and used without further purification. Distilled water was used throughout the experiment.

### 2.2. Reagents

Iron (III) solution (100 *μ*g/mL) was prepared by dissolving 0.086 g of ammonium ferric sulphate dodeca hydrate in few drops of concentrated sulphuric acid and then diluted up to the mark in 100 mL volumetric flask with distilled water. Sulfide stock solution (1000 ppm) was prepared by dissolving 0.748 g of Na_2_S*·*9H_2_O in 100 mL of water, and the solution was stored in a refrigerator. Working standards were prepared from stock solution by appropriate dilution on the day of use. Nitroso-R salt (0.1%): 100 mg of Nitroso-R salt was dissolved in 100 mL of water. Na_2_HPO_4_ (0.2 M) was prepared by dissolving 2.82 g of anhydrous Na_2_HPO_4_ in 100 mL of water. KH_2_PO_4_ (0.2 M) was prepared by dissolving 2.61 g anhydrous KH_2_PO_4_ in 100 mL of water. Disodium ethylenediaminetetraacetic acid [Na_2_EDTA] (0.01 M) was prepared by dissolving 0.372 g in 100 mL of water. Cetylpyridinium chloride monohydrate (1.39 mM) was prepared by dissolving 0.05 g of cetylpyridinium chloride in 100 mL of water. Potassium iodide (10%) was prepared by dissolving 10 g in 100 mL of water.

### 2.3. Aqueous Procedure

**Figure 1:**
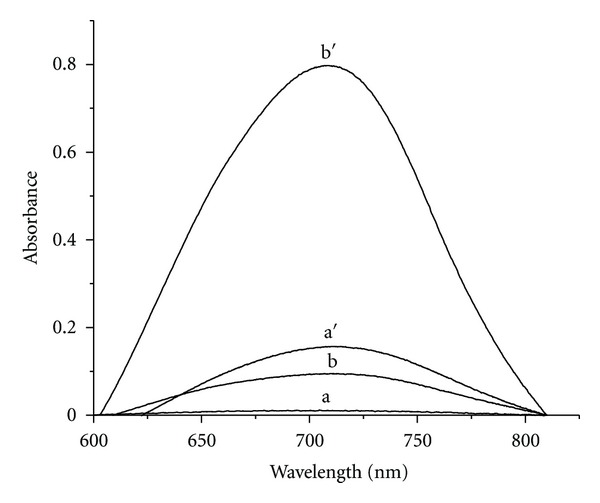
Absorption spectra. (a, b) are reagent blank and sample absorbance in aqueous phase. (a′, b′) are reagent blank and sample absorbance in micellar phase.

Aliquots (0.2–1 mL) of 10 *μ*g/mL sulfide solutions were transferred into a series of 10 mL volumetric flasks containing 1.5 mL of 100 *μ*g/mL of Fe (III) solution. Then 0.5 mL of 0.1% nitroso-R salt solution followed by 3 mL of phosphate buffer (pH 7) was added. The solutions were diluted to the mark with distilled water, and the absorbance values were measured at 710 nm (Figure 1).

### 2.4. Extraction Procedure

Aliquots (0.2–1.8 mL) of 1 *μ*g/mL sulfide solutions were transferred into a series of 10 mL volumetric flasks containing 1.5 mL of 100 *μ*g/mL of Fe (III) solution. Then 0.5 mL of 0.1% nitroso-R salt solution and 3 mL of phosphate buffer of pH 7 followed by 1.5 mL of EDTA were added, and the contents were mixed well. To that 1 mL of cetylpyridinium chloride followed by 1.5 mL of potassium iodide, solutions were added. The solutions were diluted to the mark with distilled water, and the contents were mixed well. The reaction mixture has been centrifuged for 6 min at 4000 rpm. The aqueous phase was separated by decantation process, and the micellar phase was homogenized by the addition of ethanol, and its absorbance was measured at 710 nm (Figure 1).

## 3. Results and Discussion

**Scheme 1:**
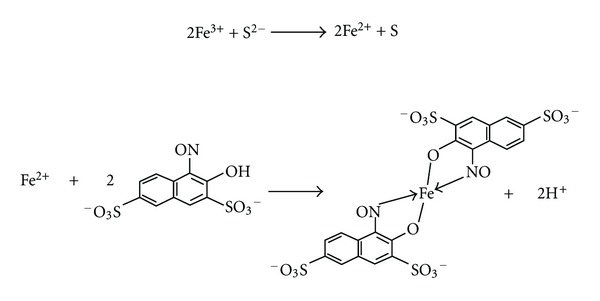
Species responsible for color formation.

The proposed method is based on the reduction of ferric ion using sulfide in mild acidic condition and the subsequent complexation of reduced ferrous ion with nitroso-R salt in alkaline medium (Scheme 1). The initial studies were carried out using 100 *μ*g/mL of iron (III), 1 mL each of 0.1% nitroso-R salt, phosphate buffer (pH 7). Absorbance values of the resulting green color complex were measured at 710 nm and correlated to the concentration of sulfide.

**Figure 2:**
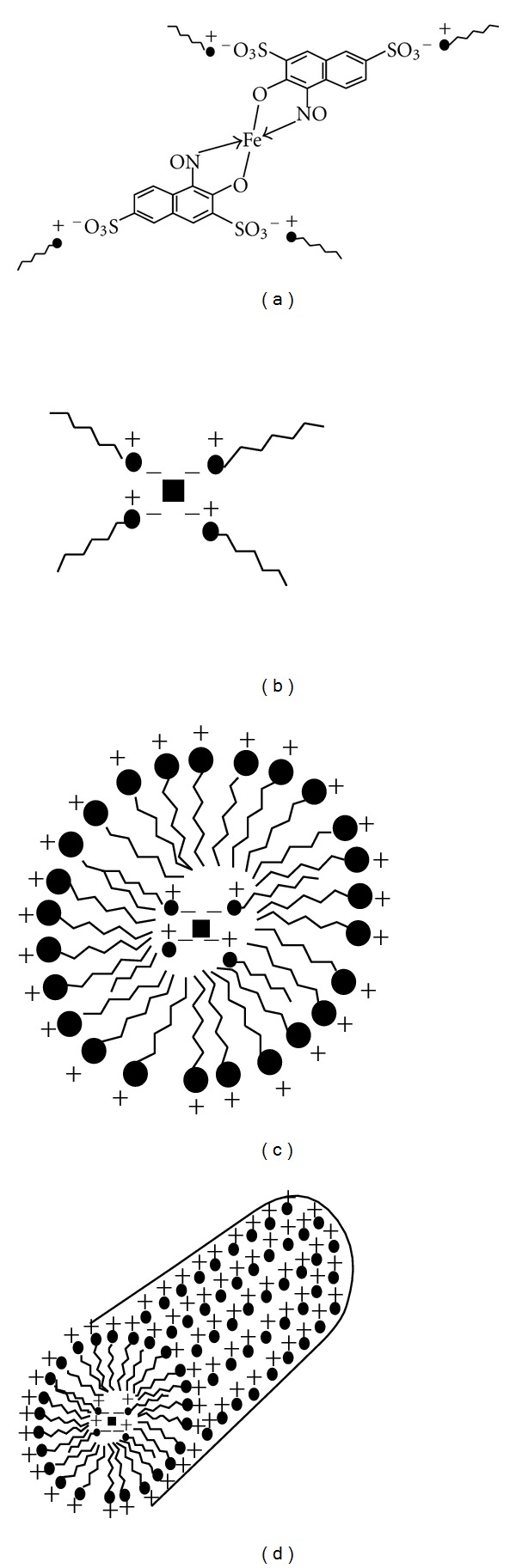
Schematic representation of metal-ligand complex at different stages in the presence of cationic surfactant. (a) Neutralized complex in bulk solution, (b) symbolic representation of electrostatically neutralized complex, (c) incorporation of metal-ligand complex into the hydrophobic core of micelle, and (d) agglomeration of the complex entrapped micelles to form the tubular or rod-like structures.

Nitroso-R salt is a sulfonated compound of 1-nitroso-2-naphthol, and its metal complexes are easily soluble in water. Because of high water solubility, the Fe (II)-nitroso-R complex cannot be extracted into any organic solvent. To improve the sensitivity and working range of the method, the cloud point extraction has been explored using cationic surfactant as a sensitizer. Cloud point extraction can be used when the target species are hydrophobic in nature. Though the Fe (II)-nitroso-R salt complex is water soluble, it has been successfully extracted into surfactant rich phase, and it can be explained through the following mechanism. When the concentration of surfactant is lower than the CMC, only slightly soluble ion associates can form between anionic ligand and surfactant monomers causing turbidity [14]. Electrostatic interaction between the metal-ligand complex and the cationic surfactant takes place through the negatively charged SO_3_^−^ group of the ligand and the positively charged head group of the surfactant molecule. The solubilizing effect of the surfactant begins at critical micellar concentration (CMC) and above, hence the neutral complexes gets trapped into the micelles (Figure 2). Once the complex gets incorporated into the micellar core, it becomes easy to separate it from the aqueous phase. Addition of salts to ionic micelle solution reduces the mutual electrostatic repulsions of charged head groups. This leads to an increased aggregation number and micellar diameter. High concentration of salt cause cationic surfactant solutions to separate into immiscible surfactant rich and surfactant-poor phases [22].

### 3.1. Optimization Study

In order to apply the developed method to quantify the trace level sulfide from a variety of environmental matrices, the reaction variables have been optimized.

### 3.2. Effect of Iron (III) Ion Concentration

**Figure 3:**
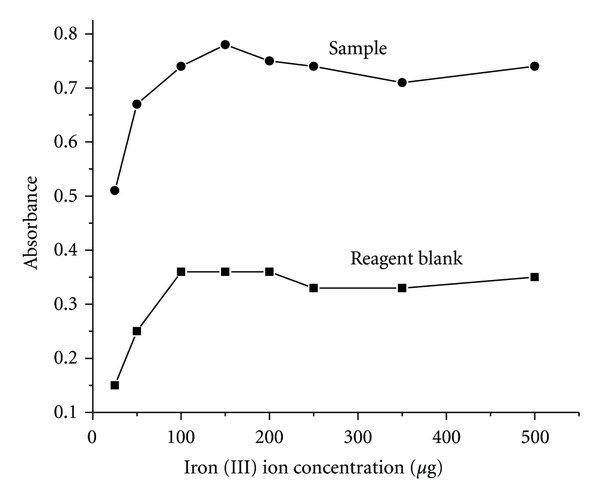
Effect of iron (III) ion.

The effect of metal ion concentration on the absorbance of the complex was studied in the concentration range of 25–500 *μ*g/mL. The absorbance of the complex Fe (II)-nitroso-R salt-cetylpyridinium chloride against reagent blank was measured at 710 nm. The absorbance value increases steadily in the concentration range of 25–100 *μ*g/mL of iron, and there after it remained almost constant as shown in Figure 3. The maximum absorbance was obtained at a concentration of 150 *μ*g/mL of iron (III), and it has been fixed as an optimum concentration and used in all further studies.

### 3.3. Effect of Nitroso-R Salt

**Figure 4:**
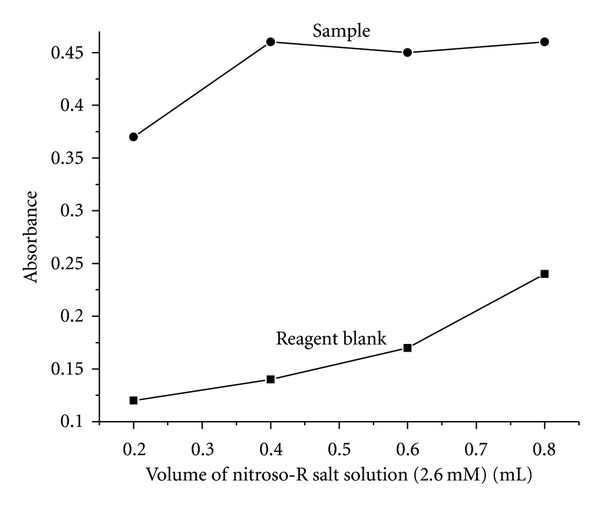
Effect of nitroso-R salt.

The effect of nitroso-R salt concentration on the extraction of the complex was investigated by varying its concentration by the addition of 0.2–0.8 mL of 2.6 mM ligand solution. The sensitivity of the method increased with the increasing volume of the ligand as shown in Figure 4. At higher concentrations, there exists a competition between ligand and its iron (II) complex to extract into the surfactant-rich phase, so the concentration of ligand in surfactant-rich phase increased. It resulted in the increase of blank value by suppressing the sample absorbance. Hence, 0.5 mL of ligand concentration has been optimized to get the maximum sample absorbance with minimum blank.

### 3.4. Effect of pH

**Figure 5:**
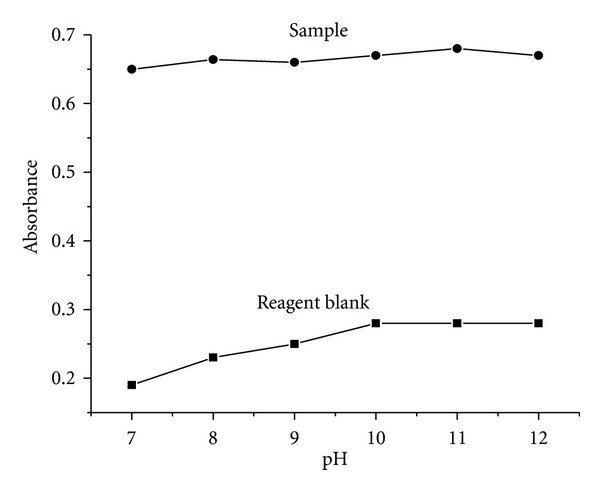
Effect of pH.

The effect of pH on the formation of metal-ligand complex was carried out in the pH range of 7 to 12 as the complexation takes place only in the alkaline condition. The variation of pH was carried out by using phosphate buffer solutions of different pH values. The sample absorbance remains almost constant throughout the pH range studied but the blank absorbance value increased with the increase in the medium pH. In order to get the low blank absorbance value, the medium pH was maintained in the range of 7-8 (Figure 5). This constant pH was achieved by the addition of 3 mL of phosphate buffer to the reaction mixture, and it was optimized and used in all further studies.

### 3.5. Effect of Surfactant

**Figure 6:**
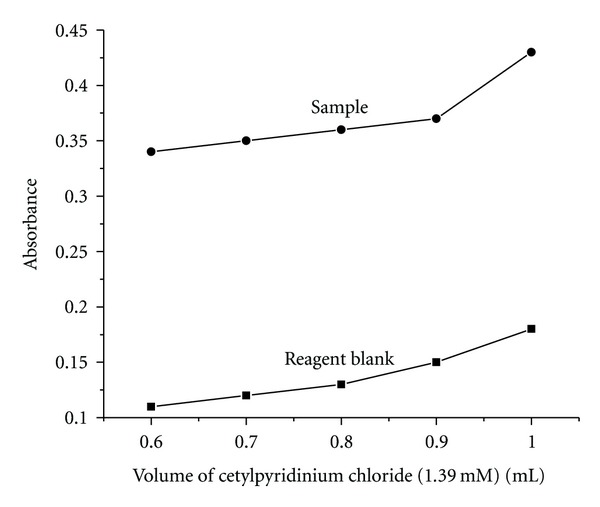
Effect of cetylpyridinium chloride.

Surfactants have been used to extract the metal-ligand complexes efficiently without using organic solvents [23]. Hence, an attempt has been made to extract the Fe (II)-nitroso-R salt complex from the aqueous solution using different types of surfactants. These surfactants are known to form aggregates which are called micelles, and these entrap the complexes very efficiently which causes phase separation. Several surfactants have been tried to separate the metal-ligand complex from aqueous phase. After adding surfactant, the solutions were heated to different temperatures to cause cloud point formation. Once clouding takes place, the phase separation can be efficiently carried out by simple centrifugation procedure. Among the surfactants used, Triton X 100 which is a neutral surfactant could not cause any quantitative extraction even after heating to 80°C. Since the complex is anionic in nature, the use of cationic surfactant may facilitate the quantitative extraction of the metal-ligand complex. Therefore several cationic surfactants were used in order to extract the metal-ligand complex quantitatively from aqueous phase. The list of the surfactants used for the extraction of the complex is listed in Table 1. Among these, only cationic surfactants could quantitatively extract the Fe(II)-nitroso-R salt complex from aqueous phase. The clouding results at room temperature itself, hence the cationic surfactants have been used in the present investigation. The monomer molecules of the cationic surfactant initially neutralize the anionic complex and form micelles. These formed micelles entrap the complex into its core and undergo clouding at room temperature (27 ± 2°C). Among the three cationic surfactants, CPC gave a higher absorbance value to the sample when compared to the other surfactants, hence CPC has been used as a surfactant in all further studies. The effect of CPC concentration on the extraction of the complex was investigated by varying its concentration by the addition of 0.6–1.0 mL of 1.39 mM solution. Extraction has not been observed below 0.6 mL of the surfactant. Extraction of the complex increased with the increase in concentration up to 1 mL; thereafter sample absorbance value almost remained constant (Figure 6). Hence, 1 mL of 1.39 mM concentration of the surfactant has been fixed as the optimum value where the absorbance of the sample was high with low blank.

### 3.6. Effect of Electrolyte

**Figure 7:**
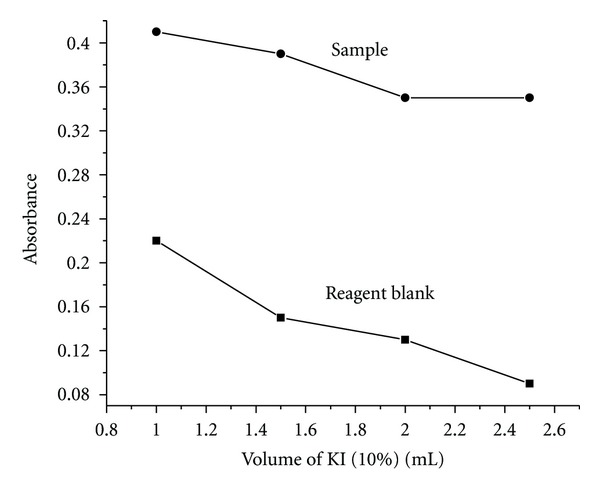
Effect of KI on the extraction of metal-ligand complex.

Separation of charged micelles from the aqueous phase can be improved by adding ionic salts like NaCl, KI, and so forth. High concentration of salt causes cationic surfactant solutions to separate into immiscible surfactant-rich and surfactant-poor phases. Different volumes, that is, 1–2.5 mL, of 10% KI solutions were used to study the effect of concentration of the salt on the extraction of the complex in the presence of cationic surfactant, cetylpyridinium chloride. No quantitative extraction was observed in the absence of KI. The complete extraction was caused with the increase in the concentration of the KI (Figure 7). There is a decrease in the absorbance values of both the blank and the sample above 1.5 mL of KI, and hence an optimum volume of 1.5 mL of 10% KI has been used in all further studies to get the low blank and high sample absorbance values.

### 3.7. Effect of EDTA Concentration

**Figure 8:**
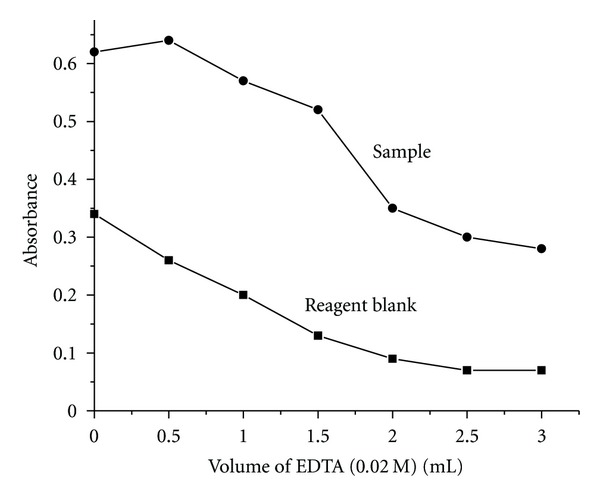
Effect of EDTA.

Addition of EDTA suppresses the blank absorbance value by masking the excess of unreacted iron (III). Therefore, EDTA was used to lower the blank absorbance value. The EDTA concentration was varied in the concentration range of 0.5–3.0 mL. In the absence of EDTA, the blank value was very high, and it decreased with the rise in the concentration of the masking agent (Figure 8). Hence, 1.5 mL of 0.02 M EDTA has been used as an optimum concentration in all further studies to obtain the maximum sample absorbance with low blank value.

### 3.8. Effect of Centrifugation Time

The effect of centrifugation time for the efficient phase separation was examined by centrifuging the resulting cloud phase at 4000 rpm at different time intervals of 2–8 min. Beyond five minutes, the absorbance values of the complex have remained constant. Hence, six-minute time has been fixed as the optimum centrifugation time.

### 3.9. Phase Diagram

**Figure 9:**
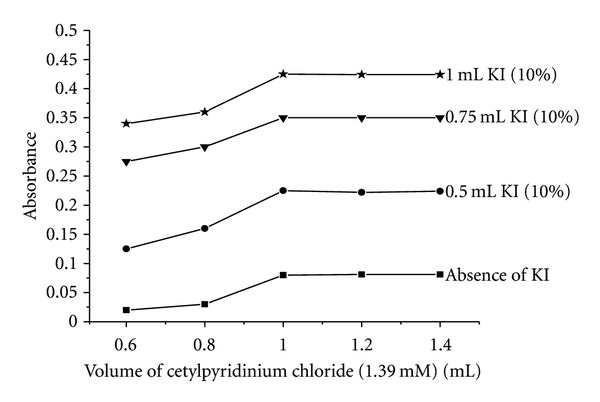
Phase diagram of the CPC, water, and KI ternary system.

The influence of electrolyte added, that is, KI [0.5 to 1.0 mL of 10%] on the extraction of Fe (II)-nitroso-R salt complex was examined at various cetylpyridinium chloride concentrations in the range from 0.6 to 1.4 mL of 1.39 mM. It is evident from Figure 9 that the extent of extraction has been increased in the presence of higher concentrations of KI. This behavior can be explained in terms of faster dehydration of surfactant molecules due to the presence of inorganic ions (here K^+^ and I^−^) competing for interaction with water molecules, that is, a “salting out effect.”

### 3.10. Analytical Figures of Merit

A simple cloud point extraction method has been developed for the quantification of dissolved sulfide using nitroso-R salt in the presence of cetylpyridinium chloride. The analytical figures such as *λ*_max⁡_, molar absorption coefficient, and detection limit of the method were found to be 710 nm, 5.66 × 10^4^, and 0.0002 *μ*g/mL, respectively. Beer's law is valid in the concentration range 0.02–0.18 *μ*g/mL of the analyte. The calibration graph has been obtained with a regression equation 0.168 + 0.500X and the correlation coefficient(R) 0.9997. The relative standard deviation has been found to be 0.0097 for 10 determinations at 1 *μ*g level. The preconcentration factor and improvement factors were found to be 5 and 5.7, respectively, in the proposed method.

### 3.11. Interference Study

The effect of foreign ions has been studied in order to apply the developed method to determine sulfide in aqueous environments. The tolerance limits of different cations and anions have been shown in Table 2. No significant interference has been observed from the common cations and anions except from sulfite and cobalt. Sulfite interfered even at 10 *μ*g/mL level due to its reducing property. The interference of sulfite can be overcome upto 100 *μ*g/mL by the addition of 1000 *μ*g of formaldehyde which forms the hydroxymethane sulfonic acid adduct. Formaldehyde did not interfere even at 1000 *μ*g level; hence it has been used as a masking agent for sulfite. Nitroso-R salt is a well-known ligand for metal ions like Fe, Co, Ni, Cu, and so forth. Cobalt forms an orange-colored complex with nitroso-R salt solution under the optimized conditions and interfered at 5 *μ*g/mL. Other metal ions like mercury, lead, and copper did not interfere. Nitrite interfered at 20 *μ*g/mL and has been overcome by adding 1 mL of 0.5% sulfamic acid up to 100 *μ*g/mL. The addition of sulfamic acid to the reaction mixture minimizes nitrite interference. Other anions like sulphate, phosphate, iodide, carbonate, chloride, and nitrate did not interfere significantly in the proposed method.

### 3.12. Application to Environmental Samples

Water passing through the landfill which collects the dissolved and suspended matter from it is called leachate. Generally, leachate may contain nitrate, phosphate, organic matter and sulfide, and so forth in significant quantities [24]. Solid wastes placed in a sanitary landfill may undergo a number of biological, chemical, and physical changes. Aerobic and anaerobic decomposition of the organic matter results in both gaseous and liquid products. Sanitary landfills are one of the important sources of groundwater contamination. Other sources include septic tanks, mining and agricultural activities, and leaking underground storage tanks. In all cases, the threat of contamination to ground water depends on the specific geological and hydrological conditions of the site. Leaking chemicals pass through the soil to the ground water system.

The developed method has been applied to determine trace level sulfide concentrations from the leachate samples as well as water samples collected from the ponds and the bore wells located near the landfills. Known quantities of sulfide were added, and the recovery studies have been carried out to validate the method.

The leachate and water samples collected were filtered using Whatman filter paper to remove any suspended and colloidal particulate matter. 20 mL of the filtered sample was taken in 25 mL volumetric flask, and 1 mL of formaldehyde (1000 *μ*g/mL) was added followed by 1 mL of sulfamic acid (0.5%). The sample was diluted to the mark, and the analysis was carried out by taking 3 mL aliquot of sample following the procedure described above. The samples were spiked with known amounts of sulfide, and the recovery of sulfide added was studied by the proposed method as well as standard method [25]. The results obtained by the proposed method are in good agreement with the standard method (Tables 3 and 4).

## 4. Conclusions

The proposed micelle mediated cloud point extraction procedure for the determination of trace quantities of dissolved aqueous sulfide is simple, sensitive, and carried out at ambient temperature using cetylpyridinium chloride as a cationic surfactant. No major interferences from the common cations and anions have been found. The proposed method does not require any organic solvent for the extraction purpose, and it has been applied to determine trace level sulfide in leachate samples and water samples. The results obtained by the proposed method are in good agreement with the standard method. It can serve as an alternative to the existing methods.

## Figures and Tables

**Table 1:** Effect of surfactant.

Surfactant type	Sample v/s blank	Temperature (°C)
	Absorbance after CPE	
None	0.08	RT
Triton X100	No extraction	80
CTAB	0.50	RT
CPC	0.56	RT
Cetrimide	0.51	RT

RT—room temperature; CTAB—cetyl trimethyl ammonium bromide, CPC—cetylpyridinium chloride.

**Table 2:** Interference study.

Interfering ion	Tolerance limit (*μ*g)
Cl^−^	>1000
SO_3_^2−^	10
SO_3_^2−a^	100
NO_2_^−^	20
NO_2_^−b^	100
NO_3_^−^	1000
SO_4_^2−^, CO_3_^2−^	>200
I^−^, PO_4_^3−^	400
Zn^2+^, Ca^2+^,	>200
Cu^2+^, Ni^2+^	25
Pb^2+^	200
Cd^2+^	50
Mg^2+^	600
Co^2+^	05
HCHO	>1000

a. Sample was treated with 1 mL of formaldehyde (1000 *μ*g/mL).

b. Sample was treated with 5 mL of 0.5% sulfamic acid.

**Table 3:** Determination of dissolved sulfide from leachate samples.

Sample	Sulfide found (ppm)		Sulfide added (ppm)	Total sulfide (ppm)		Recovery of added sulfide (%)	
	Proposed method	Standard method		Proposed method	Standard method	Proposed method	Standard method
A*	2.81 ± 0.37	2.88 ± 0.12	—	—	—	—	—
B*	1.94 ± 0.28	1.90 ± 0.15	1.0	2.86 ± 0.22	2.91 ± 0.09	99.31	102.34
C*	1.03 ± 0.21	1.05 ± 0.12	2.0	3.00 ± 0.18	3.08 ± 0.10	99.00	102.98
D*	ND	ND	1.0	1.03 ± 0.20	1.02 ± 0.09	103.00	102.00
D**	ND	ND	1.0	1.09 ± 0.18	1.02 ± 0.11	109.00	102.00

Values given here are the average of three measurements ± RSD.

ND- not detected.

*Sulfide determined by aqueous procedure.

**Sulfide determined by cloud point extraction procedure.

**Table 4:** Determination of dissolved sulfide from bore well and pond water samples using extraction procedure.

Sample	Sulfide found (ppm)	Sulfide added (ppm)	Total sulfide (ppm)	Recovery of added sulfide (%)
A^a^	0.297 ± 0.037	0.5	0.810 ± 0.033	101.6
B^a^	0.475 ± 0.028	—	—	—
A^b^	0.139 ± 0.030	0.5	0.661 ± 0.036	103.4
B^b^	0.636 ± 0.030	—	—	—

Values given here are the average of three measurements ± RSD.

^a^Water samples were collected from bore wells located near the dump sites.

^b^Water samples were collected from ponds located near the dump sites.
